# The role of deacetylase SIRT1 in allergic diseases

**DOI:** 10.3389/fimmu.2024.1422541

**Published:** 2024-07-16

**Authors:** Yun Lu, Xinyi Tang, Wenxin Wang, Jun Yang, Shengjun Wang

**Affiliations:** ^1^ Department of Laboratory Medicine, Affiliated Hospital of Jiangsu University, Zhenjiang, China; ^2^ Department of Immunology, School of Medicine, Jiangsu University, Zhenjiang, China; ^3^ Department of Laboratory Medicine, Affiliated People’s Hospital, Jiangsu University, Zhenjiang, China

**Keywords:** silent information regulator sirtuin 1 (SIRT1), deacetylase, allergic diseases, acetylation, hypersensitivity

## Abstract

The silent information regulator sirtuin 1 (SIRT1) protein is an NAD^+^-dependent class-III lysine deacetylase that serves as an important post-transcriptional modifier targeting lysine acetylation sites to mediate deacetylation modifications of histones and non-histone proteins. SIRT1 has been reported to be involved in several physiological or pathological processes such as aging, inflammation, immune responses, oxidative stress and allergic diseases. In this review, we summarized the regulatory roles of SIRT1 during allergic disorder progression. Furthermore, we highlight the therapeutic effects of targeting SIRT1 in allergic diseases.

## Introduction

The silent information regulator sirtuin 1 (SIRT1) protein is a highly conserved NAD^+^-dependent deacetylase in the sirtuin family which mainly acts as a post-translational regulator and plays a key role in histone and non-histone deacetylation. Among the sirtuins, SIRT1 was the first one discovered in mammals. The deacetylation mediated by SIRT1 profoundly impacts numerous biological processes, including DNA damage repair (1), gene transcription, glucose and lipid metabolism (2), oxidative stress (3), inflammation (4), apoptosis (5), aging (6), and autophagy (7). In addition to regulating histone acetylation, SIRT1 is also involved in many post-translational modifications of non-histones, such as transcription factors. The expression of SIRT1 was down-regulated in many acute inflammatory responses or inflammation-related diseases. SIRT1 can directly interact with or promote the histone deacetylation in the gene promoter region of inflammatory cytokines, further inhibit the transcription of target genes, and play an anti-inflammatory role, such as deacetylated HAK16 inhibits TNF-α transcription (8–10). Moreover, SIRT1 can mediate the deacetylation of inflammation associated transcription factors such as nuclear factor κB (NF-κB), activating protein 1(AP1) and HIF-1α and further decrease the expression of pro-inflammatory genes (11–15).

Hypersensitivity denotes an abnormality of immune system, contributing to hyperactive immune cells production and aggressive inflammation, which culminates in physiological disorders and/or tissue and cell damage. In recent years, the potential roles of SIRT1 in allergic diseases has been confirmed. In this review, we take deeper insights into the role of SIRT1 in hypersensitivity reactions and conclude current therapeutic avenues that target SIRT1 to alleviate these reactions.

When allergens and harmful microorganisms enter the body, epithelial cells respond by producing and releasing cytokines such as IL-25, IL-33, and thymic stromal lymphopoietin (TSLP). These cytokines activate type II innate lymphoid cells (ILC2) and contribute to the development and maturation of Th2 cells, while, dendritic cells (DCs) activate Th2 cells in the lymph nodes in a specific manner, resulting in the production of various type II cytokines, including IL-4, IL-5, and IL-13 (16–18). Upon cytokine stimulation, plasma cells secrete IgE, initiating a cascade of events within the immune system (19). IgE and the high-affinity receptor FcϵRI on the surface of mast cells and basophils put the body in a sensitized state. Upon re-exposure to an allergen, mast cells and basophils undergo activation and degranulation, thereby releasing type II cytokines that contribute to the process of allergic inflammation (20, 21). Some recent studies have observed that SIRT1 is involved in the progression of allergic inflammatory diseases. SIRT1 deacetylates transcription factors, affecting signaling pathways like AMPK, MAPK, and NF-κB, reducing inflammatory factor secretion, thereby alleviating inflammation. Targeting SIRT1 may improve clinical symptoms and tissue damage caused by allergic inflammation.

## Airway allergic inflammation

Airway Allergic Inflammation (AAI) is a chronic inflammatory disease of the airways characterized by systemic IgE elevation, eosinophil, and lymphocyte infiltration, and increased mucus secretion and airway hyperresponsiveness (AHR) (22). Eosinophil-derived pro-inflammatory mediators are a major contributor to asthma-related inflammation, which can lead to damage of airway epithelial cells, airway dysfunction, and an excess of mucus secretion (23). The aforementioned event triggers a sequence of inflammatory responses that culminate in ischemia, hemorrhage, edema, and tissue injury. Moreover, the stimulation of antigens leads to the infiltration of effector T cells into the affected tissue, thereby inducing an inflammatory reaction that can exacerbate the tissue damage.

Research has found that patients who suffer from allergic airway inflammation (AAI) may have reduced levels of SIRT1 in their lungs but high levels of SIRT1 in their peripheral blood. This correlation is positively linked to IgE levels and negatively linked to lung function (24). Therefore, measuring the level of SIRT1 in the serum could potentially assist in the diagnosis of AAI, and increasing the level of SIRT1 in the lungs may be a way to treat AAI. However, further research is needed to fully understand the connection between SIRT1 and AAI inflammation. Recent studies have shown that individuals with asthma often have increased levels of pAMPK and SIRT1 and that AMPK/SIRT1/PGC1α plays a critical role in metabolic regulation and energy expenditure during the development of AAI (25). As a result of this discovery, it has been found that the AMPK/SIRT1/Nrf2/HO-1 signaling pathway can mitigate oxidative stress in human bronchial epithelial cells and alleviate AAI (26). Additionally, researchers have noted elevated levels of IL-6 and reduced levels of SIRT1 in bronchial epithelial cells. Further studies have shown that SIRT1 inhibits Akt-dependent expression of IL-6. Consistent with this, the use of SIRT1 agonists has been found to reduce IL-6 expression and alleviate AAI (27).

AAI is an airway inflammation in which numerous immune cells are involved in disease progression. Insufficient CD4^+^CD25^+^ Tregs were found in AAI patients with impaired function, which was positively correlated with Foxo1 and SIRT1. Therefore, targeting SIRT1 may ameliorate the deficiency of Treg quantity and function and alleviate the inflammatory response (28–30). In addition, the occurrence of AAI is associated with type 3 innate lymphocyte activation, which leads to macrophage activation and the production of neutrophil chemokines. It has been found that the expression of chemokine ligand 2(CXCL2), interleukin-1β(IL-β), and tumor necrosis factor-α(TNF-α) are reduced in myeloid-specific SIRT1 deficient macrophages (BMDM). SIRT1 inhibits the ERK/p38 MAPK pathway in BMDM.SIRT1 inhibited AAI by decreasing BMDM cytokine secretion and activation of MAPK signaling pathway (31). PPAR-γ plays an important role in the inflammatory response, inducing the oxidative metabolism of macrophages by upregulating the oxidation of fatty acids and mitochondrial biosynthesis (32). PPAR-γ increased the expression of SIRT1 in macrophages and upregulated SIRT1 increased the secretion of IL-10, thus playing an anti-inflammatory role (33).

**Figure 1:**
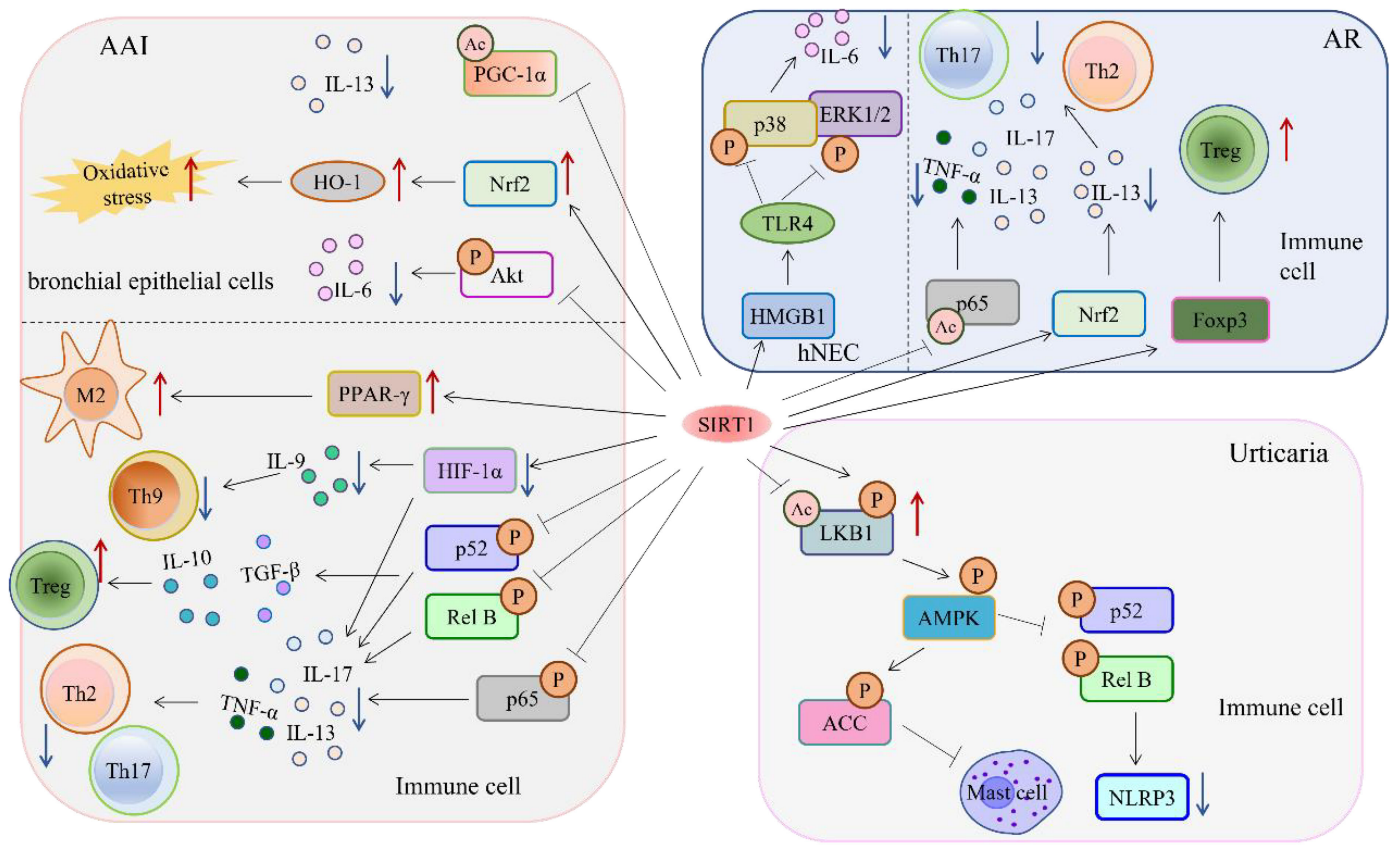
The target of SIRT1 in hypersensitivity reaction.

GATA3 and STAT6 transcription factors help Th2 cells produce type II cytokines, which can exacerbate inflammation. Low acetylation levels of GATA 3 are associated with reduced Th2 function, and SIRT1 has been reported to mediate the immune response of Th2 cells through deacetylation of GATA 3 (34–36). In autoimmune diseases and chronic inflammation, Th17 cells and their effector cytokine IL-17 are known to promote inflammation. Patients with asthma have been found to have increased Th17 cells and elevated IL-17 expression. In asthmatic mice, significant increases in eosinophils and mucus secretion were observed in their alveolar lavage fluid (BLAF). The cytokines secreted by Th2 and Th17 cells can alter the structure of bronchial epithelial cells and airway smooth muscle cells, leading to severe goblet hyperplasia and increased mucin production. NF-κB is a vital transcription factor that governs the production of pro-inflammatory cytokines and the recruitment of inflammatory cells. Additionally, it regulates the expression of several genes that are critical in the inflammatory response. In a mouse model of AAI, NF-κB has a significant impact on Th2 cell-related cytokine production and inflammatory cell recruitment. If we can reduce the phosphorylation of the classical NF-κB pathway p65 in BLAF, we may be able to decrease the production of Th2 and Th17-associated pro-inflammatory factors. This could ultimately lead to an improvement in AAI (37, 38). It is plausible to mitigate AAI by reducing the phosphorylation levels of p52 and RelB in the nonclassical pathway of NF-κB, alongside ERK1/2 and p38 in the MAPK signaling pathway (**Figure 1**). This approach can potentially lead to the reduction of Th9 cell infiltration and an elevation in the proportion of Treg (39–41). To further investigate the specific mechanisms affecting the NF-κB signaling pathway and identify therapeutic targets for the treatment of allergic asthma, the study shows that SIRT1 can inhibit the NF-κB signaling pathway and reduce the secretion of inflammatory factors, thereby controlling the inflammatory response of AAI (42–45).

The protein SIRT1 is a vital component in reducing hypersensitivity reactions by deacetylating multiple targets. Signaling pathways involved in SIRT1 hypersensitivity in different diseases. It achieves this by suppressing inflammatory factors through its ability to inhibit the NF-κB and MAPK signaling pathways. Additionally, SIRT1 activates the AMPK pathway while inhibiting mast cell activation, which works to relieve inflammation by reducing the production of inflammatory factors. SIRT1 also regulates HIF-1α activity, leading to a decrease in the proportion of Th9 cells that cause an inflammatory response. Furthermore, it regulates PPAR-γ-associated histones to enhance the immunosuppressive function of M2-like monocytes. Lastly, SIRT1 promotes the Nrf2/HO-1 signaling pathway, which supports the body’s oxidative stress response by boosting ROS production.

HIF-1α plays a vital role in the inflammatory response, and HIF-1α activation stimulates the promotion of angiogenesis, vasodilation, and vascular permeability in inflamed tissues. SIRT1 can mediate acetylation modification of HIF-1α to decrease its activity (46). SIRT1 interacts with HIF-1α to regulate immune cells. mTOR is an evolutionarily conserved serine/threonine kinase involved in cell transcription, growth, proliferation, and survival and controls cellular autophagy (47). Studies have reported that mTOR signaling mediates epithelial cell proliferation, migration, and autophagy involved in the regulation of AAI (48–50). In allergic mice, lung levels of phosphorylated (p)-mTOR were reduced, and mTOR signaling activation suppressed AAI by inhibiting autophagy (49). It has been observed that EX-527, an inhibitor of SIRT1, can enhance this phenomenon and is favorable for the control of AAI (51). mTOR acts as an upstream signal of the HIF-1α glycolysis pathway, regulating the glycolysis pathway in immune cells (52). HIF-1α is a crucial transcription factor that regulates the expression of glycolytic enzymes and plays a central role in producing pro-inflammatory cytokines (53). HIF-1α deficiency increases Treg cell production and blocks glycolysis to inhibit Th17 cell differentiation (54, 55). Recent studies have shown that SIRT1 in mouse tissue directly inactivates HIF-1α, with or without hypoxia (56, 57). Consistent with the previous results, it was found that SIRT1 deficiency-induced upregulation of HIF-1α decreased the percentage of Treg cells and increased the rate of Th17 cells. In this case, SIRT1 deficiency induced mTOR upregulation and promoted HIF-1α expression. In addition, HIF-1α not only promotes IL-9 production by regulating the glycolytic pathway in CD4^+^ T cells but also directly binds to the IL-9 gene promoter to drive IL-9 transcription (**Figure 1**). In Th9 cells, SIRT1 suppressed IL-9 transcription by inhibiting the mTOR/HIF-1α pathway, thereby alleviating AAI (58, 59).

In summary, many studies have shown that SIRT1 plays a protective role in allergic airway inflammation. Activation of SIRT1 can cause a variety of signaling pathways to promote the activation of various transcription factors, resulting in the reduction of pro-inflammatory cytokines and chemokines associated with inflammatory cells, and finally preventing or alleviating AAI. Inconsistent with the foregoing, SIRT1 has been shown to play a pro-inflammatory role in OVA-induced mouse models of airway inflammation (60, 61). This may involve SIRT1 inhibiting PPAR-γ in DC cells and promoting the differentiation of DC cells into Th2-like cells (62). Similarly, remission of airway inflammation was observed with pharmacological inhibitors of SIRT1 (60). The current understanding of the role of SIRT1 in AAI has been challenged by recent research findings, indicating a bidirectional effect. However, the specific mechanism underlying this phenomenon has yet to be fully elucidated. Further investigations are therefore warranted to clarify the reasons why SIRT1 appears to play a bidirectional role in airway inflammation.

## Allergic rhinitis

Allergic rhinitis (AR) manifests as sneezing, nasal congestion, nasal itchiness, and rhinorrhea (nasal discharge) due to IgE-mediated reactions to inhaled allergens. Rhinitis symptoms arise from inflammation in the nasal mucosa and/or sinuses. Research has shown that there is a decrease in SIRT1 expression in cases of AR. It is believed that SIRT1 controls the production of Th2 cell-related pro-inflammatory factors, which can be suppressed by proteins linked to the HMGB1/TLR4 pathway, ultimately reducing the severity of AR (63). In a study involving ovalbumin (OVA)-induced allergic rhinitis mice, researchers administered the SIRT1 agonist resveratrol (RSV). The results indicated a reduction in the expression of HMGB1 and TLR4 in the nasal mucosa following RSV treatment. Furthermore, SIRT1 expression was enhanced, leading to a mitigation of allergic rhinitis symptoms (64, 65).

Hypoxia-inducible factor 1α (HIF-1α) plays a crucial role in the progression of AR. Research indicates that inhibiting HIF-1α results in a notable decrease in eosinophils in BLAF, reduced levels of nasal mucosa and systemic Th2-related cytokines, and an improvement in AR symptoms (66, 67). Another important factor in AR is dendritic cells, which regulate T cell proliferation and differentiation through signaling (68, 69). Interaction between T-cell immunoglobulin and mucin domain 4(TIM4) and TIM1 increases PI3K/Akt phosphorylation in CD4^+^ T cells and enhances SIRT1 expression. Additionally, SIRT1 promotes Th2 CD4^+^ T cell proliferation by inhibiting Fas ligand and caspase-3 expression (70). Further exploration of how HIF-1α influences AR has revealed that HIF-1α-deficient dendritic cells utilize the SIRT1/NF-κB pathway to mitigate the inflammatory response, thus alleviating AR (71).

Recent studies have shown that beyond Th1 and Th2, Th9, Th17, and Treg play significant roles in the progression of AR (72, 73). Tregs interact directly with immune cells, releasing anti-inflammatory cytokines to maintain immune tolerance in the body. In AR, Tregs inhibit Th2 differentiation and restrict airway inflammation (74). Modulating SIRT1 levels can enhance Foxp3 expression, thereby boosting Treg function and differentiation (75, 76). Furthermore, SOCS1 modulates T cell activation, development, and differentiation by negatively impacting the Janus kinase (JAK)/STAT signaling pathway (77). Based on the research as mentioned above results, some researchers have stated that it has been observed in the progression of AR disease that inhibition of the SOCS1/SIRT1 pathway in CD4^+^ T cells promotes the proliferation of Treg, thereby inhibiting the proliferation of Th2 and alleviating AR (78). In contrast, some researchers reported that loss of Sirt1 impairs Treg survival, leading to antigen-induced T cell proliferation and inflammation (79). This difference may be caused by different disease models and research backgrounds, and the specific mechanism remains to be studied in depth.

IL-13 secreted by Th2 cells is considered to be a central mediator of allergic inflammation, stimulating mucin synthesis and secretion (80). Studies have shown that IL-13 can induce inflammatory responses and mucus secretion in human nasal epithelial cells (hNEC), and the severity of AR can be determined based on the expression of IL-13 (81). Nrf2 and Kelch-like ECH associated protein (Keap 1) combine as dimers in the cytoplasm. When stimulated, Nrf 2 dissociates from Keap 1 and translocate into the nucleus, inducing the expression of HO-1 (82). In addition, results show that activating the Nrf2/HO-1 pathway can inhibit the progression of AR (83, 84). Here, some researchers found that Formononetin relies on activation of the SIRT1/Nrf2 pathway to reduce the secretion of inflammatory factors and mucus formation caused by IL-13 (85). Consistent with this, SIRT1 mediates acetylation modification of NF-κB p65, inactivating the NF-κB pathway (86). Interfering with HDAC4 can restore the expression of SIRT1 and reduce the inflammatory response caused by IL-13 by activating the SIRT1/NF-κB pathway (81).

In conclusion, the expression of SIRT1 in nasal epithelial cells showed a protective effect on AR, and high expression of SIRT1 could reduce the inflammatory response of nasal epithelial cells and the production of mucus. In addition, during the progression of AR, the expression of SIRT1 in immune cells such as Tregs also showed protective effects on inflammation, mainly by promoting the proliferation of anti-inflammatory cells Tregs. Consistent with reports related to AAI, in AR, it is now found that SIRT1 has different effects on different immune cells, and in DCs, SIRT1 expression has been reported to show pro-inflammatory effects. SIRT1 shows different effects in different cells in the pathogenesis of AR, and the specific reasons for this are still unclear and need to be studied.

## Urticaria

Urticaria is a type of immune response that results in localized inflammation and swelling. It is caused by the widening and increased permeability of small blood vessels in the skin and mucous membranes. This condition can present as either rubella, angioedema, or a combination of both (87, 88). The sensitization of mast cells initiates the development of urticaria through IgE, which leads to the release of inflammatory mediators like histamine and other pro-inflammatory factors (87). The progression of urticaria can also be influenced by an imbalance in immune cells, particularly CD4^+^ helper T cells. This heightened sensitivity can contribute to the development of urticaria (89, 90). During the inflammatory response, JNK is activated by MEK kinase 2 (MEKK2) under the influence of inflammation and stress. Subsequently, JNK participates in the activation of mast cells triggered by antigens, which leads to the expression of TNF-α and IL-6 (91, 92). Moreover, research suggests that the level of phosphorylation of ERK is closely related to the expression of TNF-α (93). When the endogenous signal is recognized by the TLR4 receptor, IκB in the NF-κB signaling pathway phosphorylates and regulates the nuclear translocation of downstream NF-κB p65, thereby promoting the transcription of NLRP3 and IL-1β (94). It is well known that the NLRP3 inflammasome is a crucial factor in regulating apoptosis (95). NF-κB and MAPK signaling pathways can activate NLRP3 (96). According to recent reports, there is a strong correlation between NLRP3 and the onset of urticaria (97). To effectively treat this condition, it’s crucial to regulate the TLR4/NF-κB/MAPK/NLRP3 inflammasome cascade. Moreover, SIRT1 can inhibit NLRP3 activation by boosting the LKB1/AMPK pathway (98). The AMPK/SIRT1 pathway is also known to curb the activation of NF-κB/NLRP3 (99, 100). In line with these findings, recent studies have pointed out that Jingfang Granules (JFG) can increase the expression of AMPK and SIRT1 by promoting the phosphorylation of LKB1 and AMPK, thereby inhibiting the activation of NLRP3, inhibiting OVA/aluminum hydroxide-induced skin inflammation, and alleviating Urticaria disease symptoms (94).

The role of SIRRT1 in urticaria is still less studied. Since SIRT1 is a key regulatory factor in the regulation of glucose metabolism and insulin secretion, researchers have observed that SIRT1 can participate in the disorder of glucose metabolism in the skin tissue of urticaria mice, and up-regulation of SIRT1 can increase insulin secretion. Promote aerobic oxidation, inhibit glycolysis, and reduce the expression of pro-inflammatory factors, effectively relieve urticaria. Whether SIRT1 is also involved in other cellular processes in the pathogenesis of urticaria is unknown.

## Atopic dermatitis

Atopic dermatitis (AD) is a chronic skin condition characterized by persistent itching and eczema-like lesions. The development of AD has been linked to mutations in the filaggrin (FLG), as well as the presence of inflammatory factors like IL-4,IL-5and IL-13 (101). These elements can damage the skin’s barrier and trigger the migration of eosinophils (102, 103). Here, researchers investigate how SIRT1-mediated FLG or inflammatory immune cells contribute to AD inflammatory response. Studies have shown that SIRT1 levels are reduced in AD, which supports the finding that SIRT1 can help maintain the skin barrier in mouse models of AD (104, 105). Among them, FLG deficiency caused loricrin deficiency and SIRT1 pathway was destroyed (106). Deficiencies in FLG can result in a lack of loricrin and harm to the SIRT1 pathway, which is associated with inflammatory damage in AD. To combat this, targeting SIRT1 presents a potential solution. Resveratrol, an SIRT1 agonist, may serve as a therapeutic option by blocking Akt, MAPK, NF-κB, and STAT3 signaling pathways, reducing inflammatory factors, inhibiting oxidative stress and angiogenesis, and potentially alleviating the condition (107–109).

While SIRT1 levels in keratinocytes and dermal fibroblasts are upregulated, HDAC6 and CXCL13 levels are also upregulated to aggravate the inflammatory response of specific dermatitis, which is involved in the increased expression of Th1 and Th2 cytokines and the downregulation of Foxp3 and IL-10. Inconsistently, studies have suggested that SIRT1-deficient mice are sensitive to OVA’s percutaneous attack, and SIRT1 expression has a protective effect on the skin tissue of AD mice.

It has been observed that during anaphylactic shock, when SIRT1 is specifically knocked out in mast cells, there is an increase in AMPK-dependent FcϵRI signaling. This leads to an enhancement in mast cell activation both *in vitro* and *in vivo*, as confirmed by two separate studies (110, 111). At the same time, SIRT1 also weakened the inhibition of AMPK pathway through protein tyrosine phosphatase 1B (PTP1B), while also enhancing the tyrosine kinase (Syk) pathway in the spleen. As a result, it effectively inhibits allergic inflammation (111).

## Treatment

Recent reports have explored treatment options for allergic inflammation, including drugs that target SIRT1 to provide relief for allergic diseases (**Table 1**). Among the drugs that have demonstrated effectiveness in alleviating AAI are Allopurinol, GW9962, Hylocereus undatus flower (HUF), Pterostilbene (Pts), Gentiopicroside (GPS), and EX-527 (26, 33, 43, 51, 112, 113). Studies have found that SIRT1 expression in immune cells can help relieve allergic rhinitis, and the use of SIRT1 agonist resveratrol has shown promise in treating AR (64, 71). Similarly, the use of resveratrol can also alleviate AD (109). However, in nasal epithelial cells, the inhibition of SIRT1, such as with Formononetin, has been found to reduce AR, and further study is needed to understand the specific mechanism behind this (85). Studies have revealed that Jingfang granules exhibit promising outcomes in treating urticaria cases as they significantly reduce OVA/alumina-induced inflammation and lesions in mice (94).

**Table 1:** Drug therapy targeting SIRT1.

Intervention	Pathways	Effects
**Allopurinol**	SIRT1/HMGB1; SIRT1/Nrf2/ROS	Decreased PARP-1 activity enhances SIRT1 activity, which in turn leads to decreased acetylation of HMGB1 and increased Nrf2 expression, ultimately leading to decreased reactive oxygen species (ROS) content and reduced inflammation (112)
**HUF**	SIRT1/p38MAPK/NF-κB/caspase-1	The expression of p38MAPK, NF-κBp65 and caspase-1 was down-regulated while SIRT1 was up-regulated, thus inhibiting oxidative stress and inflammation (113)
**Pts**	AMPK/SIRT1/Nrf2/HO-1 pathways	Activation of p-AMPK/SIRT1 and Nrf2/HO-1 signaling pathways and inhibition of LPS-induced ROS increase in 16HBE cells (26)
**sirtinol**	SIRT1/HIF-1α	An increase in sirtuin 1 activates HIF-1α to increase VEGF expression, which promotes airway inflammation (60)
**Gentiopicroside**	SIRT1/NF-κBp65	Up-regulated SIRT1 and down-regulated NF-κB inhibited the recruitment of inflammatory cells and secretion of inflammatory factors in BALF, thus alleviating AAI (43)
**Bergenin**	SIRT1/NF-κBp65	Enhanced SIRT1 activity blocked NF signaling pathway and inhibited TNF-α-induced asthma-related AAI (45)
**EX-527**	SIRT1/mTOR/autophagy axis	Inhibition of SIRT1 activity enhanced the activation of mTOR, and then inhibited autophagy in allergic mice and inhibited AAI (51)
**SIRT1**	SIRT1/HMGB1/TLR4	SIRT1 inhibits the expression of HMGB1/TLR4 signaling pathway related proteins, reduces the production of inflammatory factors, and alleviates AR (63)
**Formononetin**	SIRT1/Nrf2	Activate the SIRT1/Nrf2 signaling pathway, thereby inhibiting IL-13 secretion, thereby reducing mucus and inflammation formation, and alleviating AR (85)
**Resveratrol**	SIRT1/HMGB1/TLR4	It inhibited the expression of HMGB1 and TLR4, promoted the expression of SIRT1, reduced histamine release, inflammatory cell infiltration, and weakened OVA-induced AR (64)
	SIRT1/Akt	Activation of SIRT1 reduces phosphorylation of Akt, leading to apoptosis and alleviating AD (109)
**Jingfang granules**	SIRT1/LKB1/AMPK	Activate the LKB1/AMPK/SIRT1 signaling pathway, inhibit the expression of inflammation-related proteins thereby alleviating skin lesions and inflammation in urticaria mice (94)
**Tan IIA**	SIRT1/LKB1/AMPK	The activation of SIRT1/LKB1/AMPK signaling pathway inhibits the activation of FcϵRі-mediated mast cells, thereby alleviating anaphylaxis (110)

HUF, Hylocereus undatus flower; Pts, Pterostilbene; Tan IIA, Tanshinone IIA.

## Conclusion

SIRT1 is a lysine deacetylase that relies on NAD^+^ to function. Its impact on the secretion of inflammatory cytokines and regulation of immune cell differentiation is mediated through the deacetylation modification of transcription factors. In recent years, the role of SIRT1 in hypersensitivity has garnered significant attention, with the exact mechanism of hypersensitivity confirmed. Consequently, we shall delve into the relationship between SIRT1 and various hypersensitivity mechanisms. SIRT1 is recognized for its anti-inflammatory properties, which have been validated in multiple hypersensitivity reactions. In mouse models, certain SIRT1-targeting drugs have exhibited potential in mitigating diseases linked to hypersensitivity reactions. This presents a promising new therapeutic avenue for treating hypersensitivity inflammatory reactions. Nonetheless, the exact correlation between SIRT1 expression and the mechanism of hypersensitivity reaction remains the subject of ongoing research. Additionally, the suitability of SIRT1-targeting drugs for clinical treatment necessitates further confirmation.

## Author contributions

YL: Writing – original draft. XT: Writing – review & editing. WW: Writing – review & editing. YJ: Writing – review & editing. SW: Conceptualization, Project administration, Supervision, Writing – review & editing.
